# Predomination of hypervirulent ST283 and genetic diversity of levofloxacin resistance in multidrug-resistant, hypervirulent Streptococcus agalactiae in Thailand

**DOI:** 10.1099/jmm.0.001970

**Published:** 2025-03-18

**Authors:** Wajeeorn Ouancharee, Anusak Kerdsin, Hien Van Doan, Chanagun Chitmanat, Kiatichai Faksri, Aroonlug Lulitanond, Aroonwadee Chanawong, Nicha Charoensri

**Affiliations:** 1 Biomedical Sciences, Graduate School, Khon Kaen University, Khon Kaen, Thailand; 2 Centre for Research and Development of Medical Diagnostic Laboratories (CMDL), Faculty of Associated Medical Sciences, Khon Kaen University, Khon Kaen, Thailand; 3 Faculty of Public Health, Kasetsart University Chalermphrakiat Sakon Nakhon Province Campus, Sakon Nakhon, Thailand; 4 Department of Animal and Aquatic Sciences, Faculty of Agriculture, Chiang Mai University, Chiang Mai, Thailand; 5 Faculty of Fisheries Technology and Aquatic Resources, Maejo University, Chiang Mai, Thailand; 6 Department of Microbiology, Faculty of Medicine, Khon Kaen University, Khon Kaen, Thailand

**Keywords:** antimicrobial resistance gene, group B *Streptococcus*, multi-host pathogen, sequence type, virulence factor

## Abstract

**Introduction**. Group B *Streptococcus* (GBS) is a multi-host pathogen causing pneumonia and meningitis in humans as well as streptococcal diseases in tilapia and mastitis in cattle. Thailand has experienced a significant increase in GBS infections that greatly impact health and economics.

**Gapstatement**. The antimicrobial resistance (AMR) and genotype data of GBS in Thailand are still limited and require further study.

**Aim**. This study aimed to describe AMR profiles and molecular characteristics, especially antimicrobial resistance genes (ARGs) and virulence factor (VF) genes of GBS in Thailand.

**Methodology**. AMR profiles of 221 GBS isolates from humans, fish and freshwater were examined. Whole-genome sequencing of 41 representative isolates was used to investigate capsular genotypes and sequence types (STs), ARGs and VF genotypes.

**Results**. All GBS isolates were susceptible to penicillin; the majority (99.1%) showed resistance to tetracycline. In addition, the rates of resistance to clindamycin, erythromycin and levofloxacin were 22.6%, 20.4% and 2.3%, respectively; multidrug-resistant (MDR) isolates (TE-E-CM and TE-E-CM-LVX) were 19.5%. Among 41 representative isolates, the dominant types were capsular genotype III (63.4%) and ST283 (43.9%). ARGs associated with resistance to tetracycline (*tetM*, *tetO* and *tetS*), erythromycin (*ermB*, *ermA*, *mefA* and *msrD*) and clindamycin (*lsaC*, *lsaE* and *lnuB*) were identified. Additionally, point mutations responsible for levofloxacin resistance, S81L in GyrA, S79F/Y in ParC and H221Y in ParE, were found. The MDR isolates belonged to various STs, predominantly clustering in capsular types III (60.0%) and Ib (30.0%). The MDR-hypervirulent ST17 and ST19 harboured multiple ARGs and mutations affecting quinolone resistance. Different VF gene patterns were found among hypervirulent STs (ST12, ST17, ST19 and ST283). Notably, a unique nt deletion [c.(1013_1020)delG] in *pilA* was found only in ST283.

**Conclusion**. This study elucidated significant antimicrobial characteristics of a substantial number of GBS in Thailand. Moreover, the distribution of the hypervirulent ST283 and the genotypes of MDR-hypervirulent GBS were first described.

## Data Availability

The whole-genome sequencing data of 41 group B *Streptococcus* isolates were submitted to the Sequence Read Archive under BioProject ID PRJNA1192395. The nt sequences of ARGs have been deposited at GenBank under the following accession numbers: *tet* genes (MZ590572–MZ590589), *erm* genes (MW602970–MW602978), *msrD* (MZ590570), *mefA* (MZ590571), *lsa* and *lnu* genes (MW602979–MW602984) and point mutations in QRDRs (MW628873–MW628879).

## Introduction

*Streptococcus agalactiae* [group B *Streptococcus* (GBS)] is regarded as a multi-host pathogen due to its ability to cause various diseases in humans, other mammals and aquatic animal species [1]. In humans, GBS is the leading cause of neonatal sepsis and meningitis and is linked to severe adult infections such as bacteremia, pneumonia and soft tissue infections [2]. Among economically important animals, such as cattle and fish, it is also a common pathogen causing mastitis in dairy cows and streptococcal diseases in farmed fish [3,4]. Consequently, GBS accounts for significant health and economic burdens. These burdens require effective management, for which comprehensive data, especially the distribution of strains and antimicrobial resistance (AMR) characteristics, are essential.

GBS exhibits diverse strain distribution, and certain strains are more prevalent and virulent than others. Given their roles in pathogenesis, capsular polysaccharide typing has been employed, and significant attention has been drawn to certain capsular types including Ia, Ib, II, III and V [5,6]. To be more precise in strain differentiation, MLST has been used, and particular sequence types (STs) have been recognized as a pathogen of great concern. These are ST1, ST10, ST12, ST17, ST19, ST23 and ST283, which are associated with severe infections both in human neonates and adults [7,9], and epidemic streptococcosis in aquaculture [3]. In addition, a recently emerging ST283 is not only a main causative agent of epidemic tilapia (*Oreochromis niloticus*) streptococcal diseases in Southeast Asia (SEA) but also a major strain causing human invasive infections [3] and severe foodborne outbreaks [10]. Recently, Schar *et al*. have reported that the emergence of ST283 is linked to the expansive growth of freshwater aquaculture production, largely in Asia including Thailand [11]. As a result, freshwater environments may serve as a key reservoir for ST283.

AMR is one of the important characteristics of bacterial pathogens. In GBS, rising rates of AMR and accumulation of antimicrobial resistance genes (ARGs) have been reported globally [12]. Penicillin (P) remains the first-line drug for the treatment of GBS infections. P non-susceptible GBS has also been discovered, but fortunately at a low rate (2.9%) from a few geographic areas only [13]. However, increased resistance rates to other relevant antibiotics including erythromycin (E) (macrolides), clindamycin (CM) (lincosamides) and levofloxacin (LVX) (fluoroquinolones) have been reported worldwide [12]. Predictably, multidrug-resistant GBS (MDR-GBS) and ARGs have also increased [12,14]. The ARGs of significant antibiotic classes included *tetL*, *tetM*, *tetO* and *tetS* for tetracycline (TE); *mefA* and *msrD* for macrolides; *lnuB*, *lsaC* and *lsaE* for lincosamides; and *ermA*, *ermB* and *ermT* for macrolide, lincosamide and streptogramin B (MLS_B_) [15]. Additionally, LVX resistance could be conferred by mutations resulting in aa substitutions in quinolone resistance-determining regions (QRDRs) of GyrA, ParC and ParE [16,18].

GBS harbours a variety of virulence factors (VFs) that are associated with various steps of the pathogenesis. These factors enable GBS to colonize host tissues [e.g. laminin-binding protein (*lmb*) and pilus island 2a (*pilA*)], function as toxins [e.g. CAMP factor (*cfa/cfb*) and *β*-haemolysin/cytolysin (*cylE*)], evade immune defences [e.g. capsular polysaccharide (*cpsA*) and C5a peptidase (*scpB*)] and promote tissue invasion [e.g. hyaluronate lyase (*hylB*) and *α*-C protein (*bca*)] [19]. VF data are also crucial for monitoring the emergence of hypervirulent strains and for the development of vaccines and other protection strategies.

Strain distribution, AMR rates, ARGs and VF profiles among GBS are varied according to several factors including geographical areas. Data of local GBS strains are therefore crucial for effective antimicrobial uses and developing tailored strategies for GBS control and management. In Thailand, although reports of GBS infection incidents have increased over the past 5 years [20,21], comprehensive documents detailing molecular types, AMR genotypes and VFs remain scarce. In this study, 221 GBS isolates from human, fish and freshwater were characterized for their AMR patterns, and 41 representative GBS isolates were investigated for their molecular characteristics via whole-genome sequencing (WGS). The distribution of AMR patterns, genotypes, ARGs and VFs of GBS in Thailand was described. To the best of the authors’ knowledge, this is the first report on the characteristics of AMR traits of GBS from humans and water, especially MDR and hypervirulent GBS strains in Thailand.

## Methods

### Bacterial isolates and species identification

**Fig. 1.**
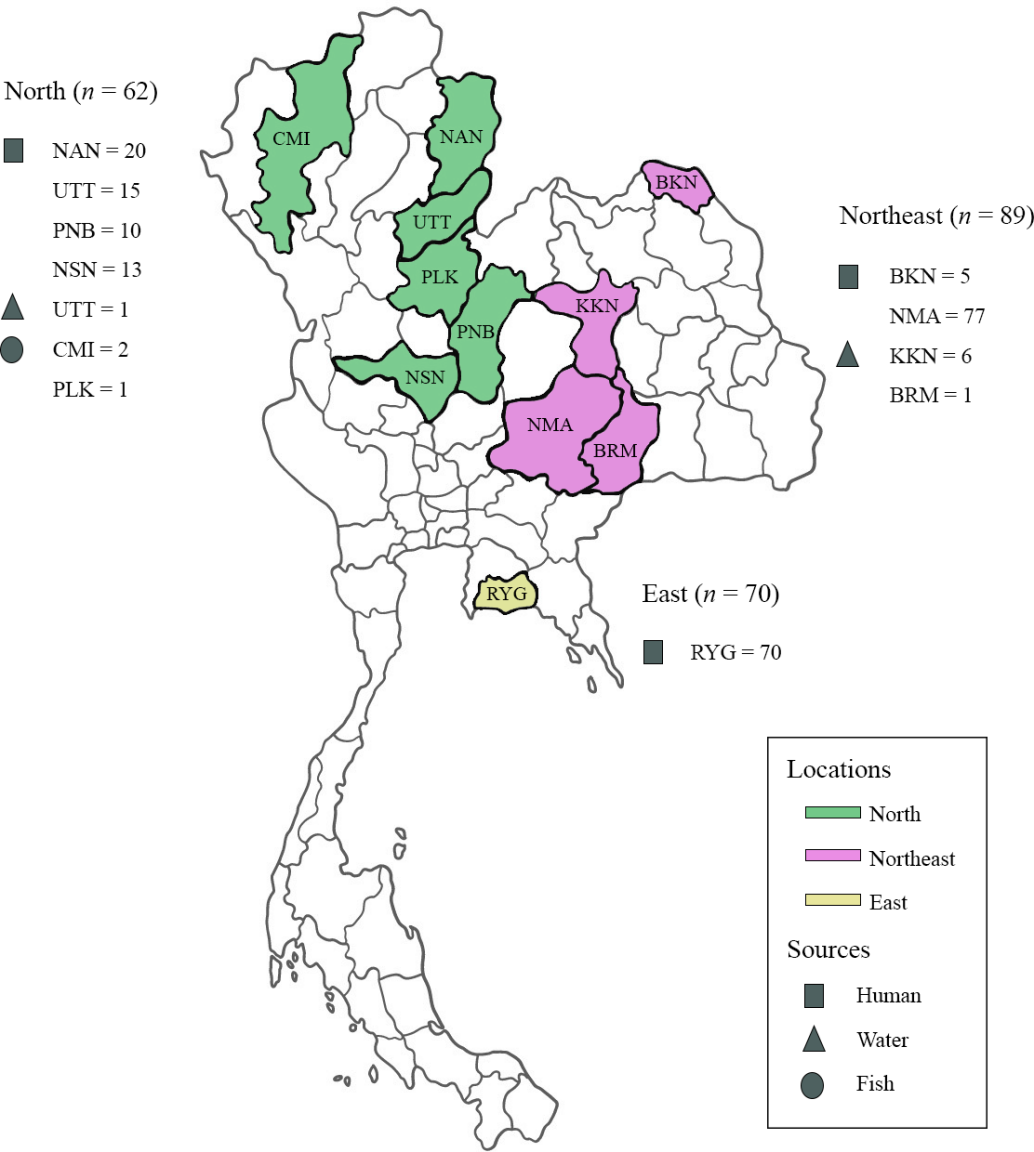
Locations and sources of GBS populations in this study (CMI, Chiang Mai; NAN, Nan; UTT, Uttaradit; PLK, Phitsanulok, PNB, Phetchabun; NSN, Nakhon Sawan; BKN, Bueng Kan; KKN, Khon Kaen; NMA, Nakhon Ratchasima; BRM, Buri Ram; and RYG, Rayong).

A total of 221 non-duplicate GBS isolates from human (*n*=210), water (*n*=8) and tilapia (*n*=3) were collected from 11 provinces in the North (*n*=6), Northeast (*n*=4) and East (*n*=1) of Thailand between November 2018 and July 2019 (Fig. 1).

The human isolates from nonredundant patients were obtained from seven provincial hospitals: Nakhon Ratchasima (*n*=77), Rayong (*n*=70), Nan (*n*=20), Uttaradit (*n*=15), Nakhon Sawan (*n*=13), Phetchabun (*n*=10) and Bueng Kan (*n*=5). Demographic data on clinical isolates such as patient age, patient gender and specimen type were provided by the hospitals. Based on specimen sources, the isolates from sterile sites including blood, cerebrospinal fluid (CSF), joint-synovial fluid, dialysis fluids, pus-internal organ and tissue were considered invasive, whereas those from pus-external organ, genital tract, urinary tract and respiratory tract were considered non-invasive.

The water isolates were screened from 164 water samples collected from swamps, wastewater, fishponds and fish tanks at fresh markets in 13 provinces across Northern, Northeastern and Eastern Thailand. Briefly, samples were spread on a selective agar, brain heart infusion (BHI, HiMedia, Maharashtra, India) supplemented with 0.5% yeast extract (HiMedia, Maharashtra, India), 8 µg ml^−1^ gentamicin (Thermo Fisher Scientific, Waltham, MA, USA) and 15 µg ml^−1^ nalidixic acid (Sigma-Aldrich, St. Louis, MO, USA). The plates were incubated in a candle jar at 37 °C for 18–24 h. GBS isolates were obtained from Khon Kaen [*n*=6: three isolates from swamps (KJN2-2, KJN2-3 and WANG2-5), two from wastewater (KSD1 and KSD18) and one from fish tank (KKN7-3)], Uttaradit [*n*=1: from fishpond (UT-SL-4)] and Buri Rum [*n*=1: from fish tank (BRM3-5)].

The fish isolates were collected from nonredundant infected Nile tilapia exhibiting clinical symptoms such as skin lesions, exophthalmia (bulging eyes), eye haemorrhages, jaw pustules and abnormal swimming. To isolate GBS from infected fish, the contents from brain, liver, spleen, posterior kidney and abscesses were grown on BHI agar and incubated in a candle jar at 37 °C for 18–24 h. Three fish isolates were obtained from cage farms in Northern Thailand: two isolates (DOI2 and DOI6) from the Ping River, Doi Lo District, Chiang Mai Province, and one isolate (PLK4) from the Nan River, Phrom Phiram District, Phitsanulok Province. These isolates were kindly provided by Associate Professor Chanagun Chitmanat, Faculty of Fisheries Technology and Aquatic Resources, Maejo University, Thailand.

Conventional biochemical tests including Gram staining, starch hydrolysis and catalase tests were performed for presumptive GBS identification. MALDI-TOF MS instrument Microflex LT/SH and Biotyper software (Bruker Daltonics, Bremen, Germany) in accordance with the manufacturer’s instructions were then used for definitive GBS identification.

### Antimicrobial susceptibility testing

Susceptibility testing was performed by the disc diffusion method according to the guidelines described by the Clinical and Laboratory Standards Institute criteria [22]. The antibiotic discs used were P (10 units), ceftriaxone (CRO; 30 µg), vancomycin (VAN; 30 µg), E (15 µg), TE (30 µg), LVX (5 µg), CM (2 µg) and linezolid (LZD; 30 µg). *Streptococcus pneumoniae* ATCC 49619 was used as an antimicrobial-susceptible control strain. GBS isolates resistant to three or more antimicrobial agents of different classes were classified as MDR-GBS.

### WGS and data analysis

Forty-one GBS isolates submitted to WGS were selected based on their AMR patterns, hosts and infections. Genomic DNA extraction was performed according to Anderson and McKay [23] and Wilson [24]. DNA concentrations and purity were evaluated using BioSpectrometer^®^ fluorescence (Eppendorf, Hamburg, Germany) and confirmed the absence of DNA degradation by agarose gel electrophoresis. Two micrograms of genomic DNA were used for WGS. The library with an insert size of 350 bp was constructed using NEBNext Ultra II DNA Library Prep Kit (New England BioLabs, Ipswich, MA, USA) and sequenced using a 150 bp paired-end Illumina HiSeq X or NovaSeq 6000 platform (NovogeneAIT Genomics Singapore Pte. Ltd., Singapore) generating more than 30× coverage. Raw paired-end reads were assembled using a *de novo* genome assembler Unicycler [25] and annotated by the RAST tool kit (RASTtk) [26] available at the Bacterial and Viral Bioinformatics Resource Center (BV-BRC v3.28.22) (https://www.bv-brc.org/).

The phylogenetic tree was analysed by the Codon Tree method based on BV-BRC PGFams as homology groups and utilized the program RAxML to analyse aligned proteins and coding DNA from 100 single-copy genes. Three reference strains – COH1 (ST17; HG939456), SG-M1 (ST283; CP012419) from GenBank and strain 8 (ST19; NMDC60045947) [18] from National Microbiology Data Center – were included in the analysis. The GBS type strain, ATCC 13813, was used as an outgroup.

### MLST and capsular genotyping

MLST and capsular genotypes were analysed based on assembled genome sequences. The seven housekeeping loci were used for MLST including *adhP*, *pheS*, *atr*, *glnA*, *sdhA*, *glcK* and *tkt* [27]. STs were determined using the *S. agalactiae* MLST online database within PubMLST (http://pubmlst.org/sagalactiae/). The capsular genotypes were analysed through two or more sequence alignments in the blastn program by comparison with the following reference sequences: accession numbers LT671983–LT671992 (http://www.ebi.ac.uk/ena/data/view/LT671983-LT671992) [28].

### Detection of AMR genotypes and virulence genes

ARGs were analysed using BV-BRC, the Comprehensive Antibiotic Research Database v3.3.0 and ResFinder v2.4.0 databases. Point mutations in QRDRs involving the *gyrA*, *gyrB*, *parC* and *parE* genes were investigated by multiple sequence alignment using clustalw2 (https://www.ebi.ac.uk/Tools/msa/clustalw2/) in comparison to GBS reference strain 2603 V/R (ATCCBAA-611; GenBank accession number NC_004116.1). VF genes were detected using VFDB (database update: 29 November 2024) and NCBI databases. Following a manual review, any gene found in the databases with at least 80.0% coverage match to the query sequence length was categorized as present.

## Results

### Demographic data of clinical GBS isolates

A total of 210 clinical GBSs were from various specimen types (Table 1). Of these, 68.6% (144 out of 210) were isolated from non-invasive infections, whereas 31.4% (66 out of 210) were from invasive infections, of which the greatest number (*n*=54) was from bloodstream infection. In addition, it should be mentioned that most of the GBSs from genital tract and urinary tract specimens (70 out of 79, 88.6%) were obtained from female patients. A unique age distribution was found in the genital tract and CSF isolates, of which were mostly obtained from young patients with the age median of 30 and 16 years, respectively. Other specimens were from patients with the age median of more than 50 years. These data indicated that our GBS population could cause various infections. Among invasive infections, bloodstream infection was prominent. The data also indicated that female is more susceptible to urinary/genital tract infections.

**Table 1.** Demographic data of the 210 clinical GBS isolates

Specimen types	No. of isolates	GenderF/M*	Age (years)Median (range)
Pus†	58	28/30	54 (14–88)
Blood	54	29/25	63 (<1–98)
Genital tract	46	46/0	30 (14–83)
Urinary tract	33	24/9	60 (<1–87)
Respiratory tract	8	4/4	53 (<1–75)
CSF	2	2/0	16 (<1–31)
Others‡	9	4/5	73 (<1–85)
Total	210	137/73	51 (<1–98)

* F, female; M, male.

† Pus specimens were from external organ (*n*=57) and internal organ (*n*=1).

‡ Others include synovial fluid (*n*=5), tissue (*n*=3) and dialysis fluids (*n*=1).

### AMR phenotypes

Of the 221 GBS isolates, only two isolates (from the urinary tract and blood) were susceptible to all tested antimicrobial agents, and 219 isolates were all susceptible to P, CRO, LZD and VAN. Resistance rates to TE, CM, E and LVX were 99.1% (219 out of 221), 22.6% (50 out of 221), 20.4% (45 out of 221) and 2.3% (5 out of 221), respectively. Six AMR patterns were observed among our GBS population (Table 2). The most prevalent pattern was TE resistance. More importantly, two MDR patterns, TE-E-CM and TE-E-CM-LVX, were found at the rates of 17.8% (39 out of 219) and 1.8% (4 out of 219), respectively. The MDR isolates were obtained from humans (both invasive and non-invasive sites) and water origins. The MDR isolates from patients with non-invasive infections gave a higher rate than those with invasive infections. In addition, the total MDR prevalence in our GBS population was 19.5% (43 out of 221). These data showed substantial rates of MDR-GBS distributed in both humans and environments.

**Table 2.** AMR patterns in the 219 GBS isolates

AMR pattern	No. of GBS isolates (%)*					
	Human			Fish	Water	Total
	Invasive(*n*=65)	Non-invasive(*n*=143)	Total(*n*=208)	(*n*=3)	(*n*=8)	(*n*=219)
TE	57 (87.7)	99 (69.2)	156 (75.0)	3 (100)	7 (87.5)	166 (75.8)
TE-E	0	2 (1.4)	2 (1.0)	0	0	2 (0.9)
TE-CM	1 (1.5)	6 (4.2)	7 (3.4)	0	0	7 (3.2)
TE-LVX	0	1 (0.7)	1 (0.5)	0	0	1 (0.5)
TE-E-CM	7 (10.8)	31 (21.7)	38 (18.3)	0	1 (12.5)	39 (17.8)
TE-E-CM-LVX	0	4 (2.8)	4 (1.9)	0	0	4 (1.8)

*The number of antimicrobial-resistant isolates included both resistant and intermediately susceptible isolates. In addition, two human isolates, each from invasive and non-invasive infections, were susceptible to all antimicrobials tested.

### Distribution of molecular types and AMR characteristics

Among the 41 GBS isolates (30 humans, 8 waters and 3 fish isolates), 8 capsular genotypes and 13 STs were identified (Table 3). Capsular genotype III was the most prevalent (26 out of 41, 63.4%) followed by capsular genotype V (4 out of 41, 9.8%). The capsular genotype III was also found predominantly among all sample groups including human invasive (10 out of 13, 76.9%) and non-invasive (6 out of 17, 35.3%) specimens, water (7 out of 8, 87.5%) and fish (3 out of 3, 100.0%) samples. In terms of ST, ST283 was the most frequent (18 out of 41, 43.9%) and present in the isolates with capsular genotype III only. It is worth to state that eight of nine (88.9%) human ST283 isolates were from invasive infections. In addition, this ST was found in all fish samples. This finding indicated that the ST283 population in this study is prone to be virulent to humans and fish. Moreover, six of eight isolates from water were ST283. This showed that ST283 was also efficiently distributed in our environments. AMR characteristics of all ST283 isolates were demonstrated to be TE^I^, and no resistance to other antimicrobial agents tested was found.

**Table 3.** Sources, molecular types and AMR characteristics in the 41 GBS isolates

Infections (*n*)	Specimens (*n*)	Capsular type (*n*)	ST (*n*)	AMR characteristics			
				Phenotype*	Genotype		
					TE	E-CM	LVX (GyrA/ParC/ParE)
**Human invasive (13)**	Blood (12)	Ia	103	TE^R^, E^I^, CM^I^	*tetS*	–	–
		Ib	12	TE^R^, E^R^, CM^R^	*tetO*	*ermB*	–
		III (9)	283 (7)	TE^I^ (6)	–	–	–
			17	TE^R^, E^R^, CM^R^	*tetO*	*ermB*	–
			19	TE^R^, CM^R^	*tetM*	*lsaC*	–
		V	1	TE^R^	*tetM*	–	–
	Joint fluids	III	283	TE^I^	–	–	–
**Human non-invasive (17)**	Genital tract female (10)	Ia (2)	314	TE^R^	*tetM*	–	–
		Ib (2)	1	TE^R^, E^R^, CM^R^	*tetM*	*ermB*	–
			10	TE^I^, E^R^, CM^R^, LVX^R^	–	*ermB*	Ser-81→Leu/Ser-79→Phe
		III (5)	17 (2)	TE^R^, E^R^, CM^R^, LVX^R^	*tetO*	*ermB*	Ser-81→Leu/Ser-79→Tyr
				TE^R^, E^R^, CM^R^	*tetO*	*ermB*	–
			19	TE^R^, E^R^, CM^R^, LVX^R^	*tetM*	*ermB-msrD-mefA-lnuB-lsaE*	Ser-81→Leu/Ser-79→Tyr/His-221→Tyr
			861	TE^R^, E^I^, CM^R^	*tetO*	*ermA-lsaC*	–
			1167	TE^R^, CM^R^	*tetO*	*lsaC*	–
		V	1	TE^R^	*tetM*	–	–
	Pus (4)	III	283	TE^I^	–	–	–
		IV	2	TE^I^	–	–	–
		V	1	TE^I^	–	–	–
		VI	1	TE^I^	–	–	–
	Urinary tract (3)	II (2)	1	TE^R^	*tetM*	–	–
			28	TE^R^	*tetM*	–	–
		IX	153	–	–	–	–
**Water (8)**		III (7)	19	TE^R^, E^R^, CM^R^	*tetO*	*ermB-lsaC*	–
			283 (6)	TE^I^	–	–	–
		V	1	TE^R^	*tetM*	–	–
**Fish (3)**		III (3)	283	TE^I^	–	–	–

* I, intermediately susceptible; R, Resistant

Besides ST283, other STs found in the capsular genotype III group were ST17 (*n*=3), ST19 (*n*=3), ST861 (*n*=1) and ST1167 (*n*=1). Interestingly, isolates of ST17 (*n*=3), ST19 (*n*=2) and ST861 (*n*=1) were MDRs of TE-E-CM (*n*=4) and TE-E-CM-LVX (*n*=2) phenotypes. In addition, MDRs with TE-E-CM were observed in other STs including ST1, ST12 and ST103 isolates, whereas the TE-E-CM-LVX resistance pattern was seen in the ST10 isolate. To be concluded, MDRs were found in seven STs including ST1, ST10, ST12, ST17, ST19, ST103 and ST861. It should be emphasized that MDR isolates with LVX resistance were distributed in ST10, ST17 and ST19, of which all were isolated from genital tract samples of female patients in Rayong Province.

These data showed that the capsular genotype III/ST283 strains were the most virulent in our GBS population and the most widely distributed in our environment. Additionally, MDR-GBSs were distributed in various genetic backgrounds of our GBS population.

### ARGs of GBS in Thailand

Resistance genes or genetic changes corresponding to TE, E, CM and LVX resistance of our GBS strains are shown in Table 3. Three types of *tet* genes corresponding to TE resistance consisted of *tetM* (*n*=10), *tetO* (*n*=7) and *tetS* (*n*=1), whereas no *tet* gene was found in any TE^I^ isolate. Each *tet* gene was distributed in different molecular types: *tetM* in ST1 (*n*=5), ST19 (*n*=2), ST314 (*n*=2) and ST28 (*n*=1); *tetO* in ST17 (*n*=4), ST12 (*n*=1), ST861 (*n*=1) and ST1167 (*n*=1); and *tetS* in ST103 (*n*=1) only.

For macrolide and/or lincosamide and MLS_B_ resistance, there were five genetic patterns: *ermB* (*n*=6), *lsaC* (*n*=2), *ermA-lsaC* (*n*=1), *ermB-lsaC* (*n*=1) and *ermB-lnuB-lsaC-msrD-mefA* (*n*=1). The most prevalent pattern, *ermB*, was found in ST1, ST12 and ST17 isolates. In addition, the *mreA* gene was identified in all 41 GBS isolates, but it did not confer an E resistance phenotype.

Aminoglycoside resistance genes including *ant(6)-Ia* and *aph(3')-III* were detected in ST12, ST17 and ST19, whilst the aminoglycoside bifunctional resistance gene [*acc(6')-Ie-aph(2'')-Ia*] and the nucleoside resistance gene (*sat-4)* were present in the ST19 isolate only (Table S1, available in the online Supplementary Material). Despite the genetic presence, phenotypic resistance to aminoglycoside and nucleoside antibiotics was not examined. An interesting pattern, *tetM-ermB-lnuB-lsaC-msrD-mefA-ant(6)-Ia-aph(3')-III-aac(6')-Ie-aph(2'')-Ia-sat-4*, was found in an ST19 isolate with LVX-R phenotype.

Only three out of five LVX-R isolates (isolate names: RYG66, RYG73 and RYG82) were genetically characterized. The STs of these isolates included ST10 (RYG66), ST17 (RYG73) and ST19 (RYG82). The analysis results showed that each LVX-R isolate contained unique LVX-R traits. The details of mutations in QRDRs of GyrA, ParC and ParE are shown in Table 3. It might be worth to mention that the RYG82 (ST19) isolate with the TE-E-CM-LVX phenotype contained the greatest number of genetic changes involved in E, CM and LVX resistance. All LVX-R isolates were from only one province, Rayong, located in the eastern part of Thailand.

### Distribution of VFs in hypervirulent clones

**Fig. 2.**
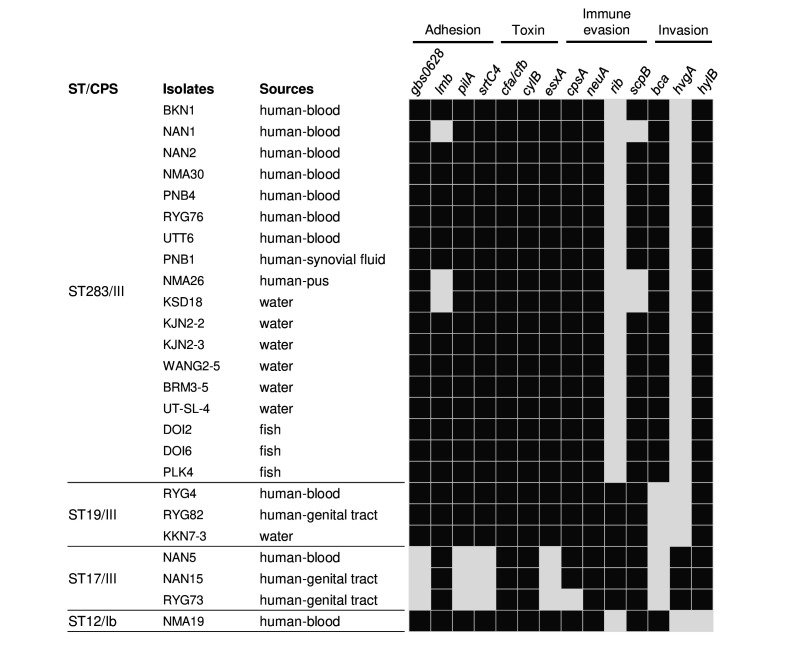
Virulence gene patterns of GBS from humans, fish and water. The presence (black) or absence (grey) of genes associated with adhesion, toxin, immune evasion and invasion is shown.

Four hypervirulent clones, including ST283 (18 isolates), ST19 (3 isolates), ST17 (3 isolates) and ST12 (1 isolate), were identified in our 41 GBS isolates. Fourteen virulence genes linked to adhesion, toxin, immune evasion and invasion were investigated in these hypervirulent isolates (Fig. 2). All isolates shared common genes including *cfa/cfb*, *cylB* and *neuA*; all ST19 and most ST283 contained the highest number of VF genes (12 of 14). Among the ST283 isolates, two VF gene patterns were identified. The majority, 83.3% (15 out of 18), carried the genes *gbs0628*, *lmb*, *pilA*, *srtC4*, *cfa/cfb*, *cylB*, *esxA*, *cpsA*, *neuA*, *bca*, *scpB* and *hylB*. The remaining 16.7% (3 out of 18) lacked the *lmb* and *scpB* genes but retained the other genes.

Additionally, the number of *esxA* genes varied among STs. All ST283 and ST12 isolates harboured four *esxA* homologues. In contrast, ST19 contained only one *esxA*, whilst no *esxA* was detected in ST17. Notably, the *rib* gene appeared exclusively in ST17 and ST19, whilst the *hvgA* gene was found only in ST17.

Interestingly, the *pilA* of ST283 exhibited an nt deletion (G) at a position between 1013 and 1020 [c.(1013_1020)delG]. This mutation introduced a premature stop codon, truncating the protein to 342 aa. In contrast, the *pilA* of a reference sequence (EU929251.1), which shares 99.9% similarity with our ST283, consists of 2691 bp and encodes a protein of 896 aa. In addition, this c.(1013_1020)delG was also identified in ST283 isolates from other countries, such as Malaysia (SA2BKE; CP168323.1), Singapore (SG-M1; CP012419.2), Hong Kong (CU_GBS_08; CP010874.1) and Brazil (S73; CP030845.1).

### Phylogenetic tree of GBS

**Fig. 3.**
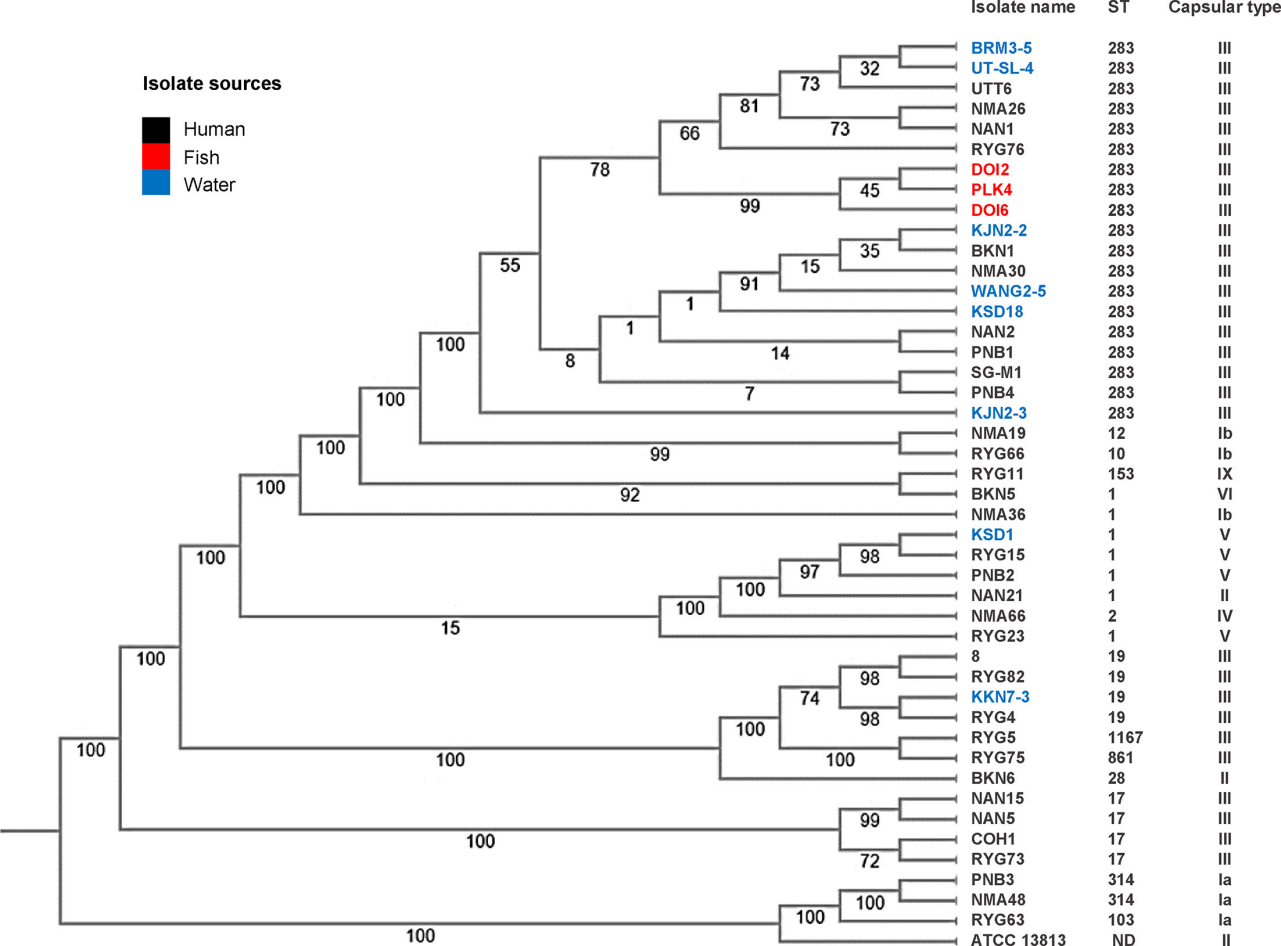
Phylogenetic tree of the 41 GBS genomes with the reference strains: ST17 (COH1), ST19 (8) and ST283 (SG-M1). The numbers at the branch points indicate bootstrap values. The *S. agalactiae* ATCC 13813 was a type strain used as an outgroup. nd is not defined.

The phylogenetic analysis revealed that the 41 GBS isolates were categorized into seven clusters and two singletons, supported by bootstrap values of ≥70 (Fig. 3). Most ST283 isolates formed a distinct cluster, suggesting a high degree of genetic similarity within this ST. However, human and water isolates were more closely related than those from fish, with intermixing in certain groups, indicating potential human-to-water transmission. Furthermore, the fish isolates showed a closer relationship within a specific group, pointing to a shared ancestral origin.

Other STs were distributed across the tree. ST1 showed high genetic diversity, being grouped into two clusters and one singleton. ST17 formed a distinct cluster and was closely related to the hypervirulent isolate COH1. Meanwhile, ST19, including MDR-RYG82, was closely related to MDR-8 (ST19) from China, which is consistent with their ARG patterns.

## Discussion

GBS is a significant pathogen threatening the health and economy of several countries, including Thailand. In humans, a population-based study of bloodstream infection in rural Thailand, during 2007–2014, showed that GBS was the eighth (~3.0%) bacterial pathogen [29]. Another study on community-acquired acute bacterial meningitis in adults during January 2002 to December 2016 at Siriraj Hospital, Thailand’s largest national tertiary referral centre, revealed that GBS was the most prevalent [21]. In another aspect, a collection of 1736 GBS isolates from patients throughout Thailand were analysed, and the common invasive GBS infections were sepsis, meningitis and septic arthritis, whilst non-invasive GBS was urinary tract infection [30]. GBS is also a main agent causing severe infections in aquaculture tilapia between 2003 and 2011 [31] and during 2012–2014 [32,33] in different areas of Thailand. In another economic animal, dairy cow, GBS was a major causative agent of bovine mastitis during 2016–2019 [4,20]. To summarize, our data, in concordance with the previous studies, pointed out that GBS is a significant threat causing health and economic burden in Thailand.

Based on capsular polysaccharide types, five genotypes, III, V, Ia, Ib and II, were found with different degrees. Importantly, we found capsular genotype III as the most predominant among our GBS population (64.3%) and among the invasive isolates (76.9%), followed by genotype V (9.8%). Our findings were in accordance with the other study in Thailand, which documented that genotype III was the most common capsular type (46.4%), followed by type V (21.0%) [30]. Our trend was also in agreement with reports from other countries in SEA, where capsular type III was the most prevalent among invasive isolates ranging from 32.2% to 77.9% [34]. Regarding strain distribution in fish, since 2012, serotype III has overcome serotype Ia [33]. In our study, only three isolates were characterized, and all were type III.

Among our GBS isolates, the ST283, regarded as a hypervirulent clone, was clearly predominant (34.3%, 12 out of 35) in human and fish isolates, similar to previous studies in Thailand [9,35] and other countries in SEA [3]. In accordance with the findings of ST283 in the genitourinary and intestinal tracts of healthy individuals [36], our results showed that ST283 was found as a major clone in water samples. Evidence from our study suggested that ST283 exhibits adaptability to a wide range of conditions including humans, fish and environment such as wastewater, swamps and fishponds. The adaptability to diverse aquatic habitats could give an opportunity in exposing to fish populations. Moreover, the presence of ST283 in the environment is important information adding to the impact of ST283 on One Health concern.

AMR characteristics of GBS in this study were in concordance with previous studies in Thailand and other countries in SEA. All isolates were susceptible to P, the first-line treatment for GBS infections, which is encouraging data. Moreover, this GBS population was all susceptible to CRO, LZD and VAN, corresponding to previous studies conducted in Thailand [37] and other countries in Asia [38,39]. In contrast, the TE resistance rate in our study (99.1%) is higher than the previous report (41.3%) studied in invasive GBS among non-neonatal patients during January 2017 to December 2018 in Thailand [40]. Nonetheless, high rates were also observed in our surrounding countries such as Indonesia (89.0%) [39] and China (93.5%) [41]. Resistance rates to CM (22.6%) and E (20.4%) were similar to those of blood isolates reported in Thailand [40]. In contrast, higher resistance rates (more than 50.0%) were reported from Vietnam [42] and China [41] during 2013–2020. LVX resistance was first reported (1.8%) from Thailand during 2017–2018 [40]. In this study, the rate of 2.3% was observed, similar to those observed in Taiwan and Indonesia [39,43], but lower than those reported from Korea (32.4%), China (37.7%) and Japan (40.2%) [2,16, 17]. In addition, 19.5% of our GBS isolates were MDR (TE-E-CM and TE-E-CM-LVX), consistent with previous findings in Thailand [40]. Notably, most genital isolates were identified as MDR, raising concerns about the potential transmission of MDR-GBS from mother to newborn. As a result, prevention measures, including prenatal GBS screening and the administration of prophylactic antibiotics, should be emphasized.

Several ARGs detected in our GBS population included *tetM*, *tetO*, *tetS*, *ermA*, *ermB*, *mefA*, *mreA*, *msrD*, *lnuB*, *lsaC*, *lsaE*, *ant(6)-Ia*, *aph(3')-III*, *aac(6')-Ie-aph(2'')-Ia* and *sat-4*. For TE resistance, *tetM* was the most prevalent, consistent with previous reports [15]. Our finding showed the absence of *tetM* in ST283, aligning with the recent reports that noted the loss of *tetM* during the evolution of this strain, especially isolates from Thailand [11,44]. However, unlike Schar *et al.*’s findings, *mreA* remained present in all ST283 isolates. Similar to this study, previous research indicated that despite the widespread presence of *mreA* among ST283 isolates, no macrolide resistance was observed [45]. In addition, our finding showed that *ermB* was strongly related to phenotypic macrolide and lincosamide resistance, supporting previous reports [15,18]. We also found *lsaC* responsible for the lincosamide resistance phenotype, similar to another report from Norway [46], but different from that (*lnuB*) described in Iran and Korea [47,48].

aa substitution pattern that conferred LVX resistance in ST10 (RYG66) was similar to those reported from several countries such as China [16,49], Japan [50] and Taiwan [43], whilst a mutation pattern in ST17 (RYG73) was concordant with the Chinese strain [49]. More interestingly, a similar pattern of the triple mutations in ST19 (RYG82) was found in Korean [17] and Chinese [16] GBS. In particular, ST19 (RYG82) harbouring multiple ARGs, including *tetM*, *ermB*, *msrD*, *mefA*, *lnuB*, *lsaE ant(6)-Ia*, *aph(3')-III*, *aac(6')-Ie-aph(2'')-Ia* and *sat-4*, exhibited the most similar AMR phenotype and genotype to the Chinese isolate [18]. The mechanism of ST19 (RYG82) genomes containing these multiple ARGs may be mediated by integrative and conjugative elements, transposons or insertion sequences reported previously [49,51].

This study also reported key VF genes associated with GBS hypervirulent clones clustered in ST283, ST19, ST17 and ST12 [3,52,54]. The VFs of ST283 identified in our study largely align with those reported by Schar *et al.* [11]. However, some differences were observed, particularly regarding *esxA*, which was detected in all ST283 isolates. The *esxA* gene encodes ESAT-6, a pore-forming protein in GBS [55]. We found that ST283 had two adjacent *esxA* homologues, consistent with the findings of Spencer *et al.* [55]. Additionally, we observed the absence of the *lmb* and *scpB* genes in human and water isolates but not in fish isolates. This finding differs from the report from Malaysia, which found the loss of *lmb and scpB* in the majority of fish isolates [56], whilst these genes remained present in human isolates, consistent with the findings by Schar *et al.* [11]. However, our study is supported by Sirimanapong *et al.*, who reported that *lmb* and *scpB* were predominantly present in fish isolates from Thailand, even though these genes were absent in Vietnamese isolates [44]. Interestingly, the deletion mutation [c.(1013_1020)delG] in the *pilA* gene of ST283 is being documented for the first time in our study; thus, this raises a question of the pathogenic roles of the mutant protein product.

Furthermore, we detected *bca* in ST283 and ST12, whilst *rib* was exclusively found in ST17 and ST19, consistent with previous studies [57,58]. In ST17, we found *hvgA*, a key factor leading to intestinal colonization, which can promote central nervous system invasion by enabling the transcellular crossing of the choroid plexus [59]. Our findings highlight the genetic diversity of VFs across different GBS STs, supporting the necessity for continued surveillance to track the evolution of hypervirulent clones and their ability to persist in both human and environmental reservoirs.

In our study, the distribution and connections of GBS strains were investigated. It was revealed that certain GBS strains were distributed across various regions of Thailand, including the North, East and Northeast. Notably, ST283 was detected in all provinces. We found that samples from humans and environmental water sources were closely related, suggesting that human contamination may spread to the environment and fish, aligning with previous reports [11]. The close relationship seen within the fish isolates remains inconclusive in this study because of the limited sample size. Furthermore, although the fish isolates were collected from different geographic locations, the rivers used for fish farming in both areas are interconnected. This information suggests that the fish isolates may originate from the same clone, making this finding unsurprising. To draw definitive conclusions on this issue, future studies should involve more extensive sampling of fish isolates from a wider range of locations. Another limitation of this study is the absence of sampling from the central and southern regions of Thailand. However, the samples during 2012–2016 from Bangkok and nearby provinces, the central region of Thailand, were characterized [9]. Therefore, further studies should include additional samples from southern regions of Thailand.

In summary, our study provided the data of AMR phenotypes and genotypes, molecular type distribution and VFs among invasive and non-invasive, water and fish GBS in Thailand. Importantly, this is the first report on molecular types of a large number of the GBS isolates in Thailand, relating to their AMR and VF genotypes. The predominance of hypervirulent ST283 and the emergence of MDR-GBS isolates in human infections, particularly those identified as ST12, ST17, and ST19, highlighted the potential health threats posed by these hypervirulent strains. Our data contribute to a better understanding of the situation of GBS in One Health, which requires regular surveillance, facilitates early intervention and performs effective prevention and control strategies.

## supplementary material

10.1099/jmm.0.001970 Uncited Table S1.

## Abbreviations

AA: amino acid
AMR: antimicrobial resistance
ARGs: antimicrobial resistance genes
BHI: brain heart infusion
BV-BRC: Bacterial and Viral Bioinformatics Resource Center
CM: clindamycin
CRO: ceftriaxone
CSF: cerebrospinal fluid
E: erythromycin
GBS: group B *Streptococcus*
I: intermediately susceptible
LVX: levofloxacin
LZD: linezolid
MDR: multidrug-resistant
MLS_B_: macrolide, lincosamide and streptogramin B
NT: nucleotide
P: penicillin
QRDRs: quinolone resistance-determining regions
R: resistant
SEA: Southeast Asia
STs: sequence types
TE: tetracycline
VAN: vancomycin
VF: virulence factor
WGS: whole-genome sequencing
